# Small Cell Carcinoma of the Prostate: A Case Report and Review of the Literature

**DOI:** 10.7759/cureus.7074

**Published:** 2020-02-22

**Authors:** Rahul Bhandari, Toms Vengaloor Thomas, Shankar Giri, Pallatikurthi P Kumar, Celeste Cook-Glenn

**Affiliations:** 1 Radiation Oncology, G.V. (Sonny) Montgomery VA Medical Center, Jackson, USA; 2 Radiation Oncology, University of Mississippi Medical Center, Jackson, USA; 3 Pathology, G.V. (Sonny) Montgomery VA Medical Center, Jackson, USA

**Keywords:** small cell carcinoma of the prostate, prostate cancer, neuroendocrine carcinoma of prostate

## Abstract

Small cell carcinoma of the prostate (SCCP) is a rare malignancy that is considered a lethal entity of prostate cancer. Once it is diagnosed, patients characteristically experience an aggressive clinical course with poor overall survival rates, which unfortunately still holds even with modern treatments. In this report, we discuss the case of a 63-year-old African American male who initially presented to the hospital with an elevated prostate-specific antigen (PSA) level of 9.41 ng/mL and was found to have locally extensive SCCP. After one cycle of chemotherapy, the patient's symptoms worsened, and his disease continued to progress with an increased metastatic burden. In a matter of just a few months, the patient’s disease progressed from a locally advanced entity to a diffusely metastatic one, showcasing the true aggressive nature of this disease. Through an extensive literature review, this case report also sheds further light on SCCP's histological characteristics, its apparent differences from adenocarcinoma of the prostate, and its aggressive nature even through treatment.

## Introduction

A subset of neuroendocrine cancer, small cell carcinoma of the prostate (SCCP) is a rare malignancy that affects <1% of the population and is considered a lethal entity of prostate cancer. It has a median survival rate of 1-2 years from the time of diagnosis. Similar to what is seen in small cell carcinoma of the lung (SCLC) or other small cell primaries, SCCP is defined by a primary tumor of the prostate gland that expresses small cell morphology and embodies high-grade features. These include deficient prominent nucleoli, necrosis and apoptosis, high nuclear-to- cytoplasmic ratio, variable tumor giant cells, positive crush artifact, high mitotic rate, and nuclear molding [1].

Diagnostic accuracy for SCCP poses challenges as its clinical and histological features are only now starting to gain recognition [2]. Once it is diagnosed, patients characteristically experience an aggressive clinical course with poor overall survival, which unfortunately still holds true even with modern treatments. 

In approximately 50% of the cases, SCCP will reveal itself as a de novo malignancy at the time of diagnosis with pure malignant neuroendocrine cells. However, in the rest of the cases, it will present in the shadow of a previously diagnosed or concomitant prostate adenocarcinoma, with distinctly differentiated histological features supporting both types. We discuss the case of a patient who presented with no symptoms at the time of diagnosis but then quickly developed a progressive and aggressive disease that was unresponsive to interventions.

## Case presentation

A 63-year-old African American male initially presented to the hospital with an elevated prostate-specific antigen (PSA) level of 9.41 ng/mL. The PSA from a year prior was normal at 0.80 ng/mL. The patient’s symptoms included occasional dysuria and infrequent nocturia. The patient denied any other lower urinary tract symptoms, hematuria, abdominal or pelvic pain, bowel abnormalities, hematochezia, weight loss, night sweats, or loss of energy. The digital rectal exam at the time revealed a large volume prostate with induration along the left side of the gland.

**Figure 1:**
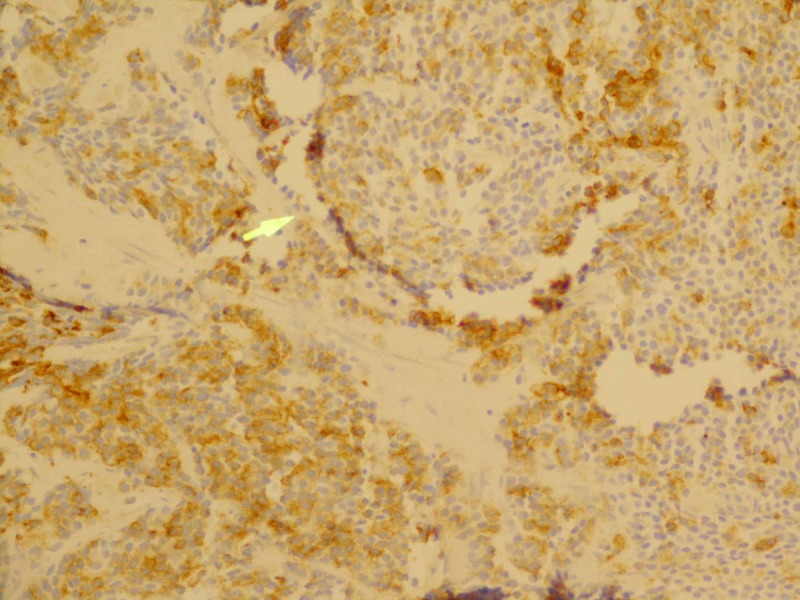
Typical punctate immunostaining pattern with low molecular weight cytokeratin (200x magnification) Yellow arrow: low molecular weight cytokeratin with the classic small cell cytoplasmic punctate staining pattern

**Figure 2:**
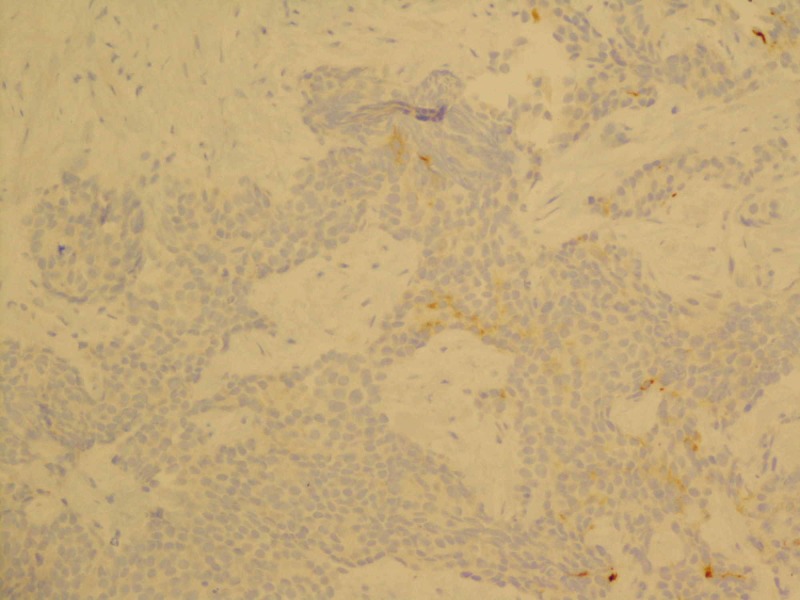
Focally positive immunostaining for synaptophysin (200x magnification)

**Figure 3:**
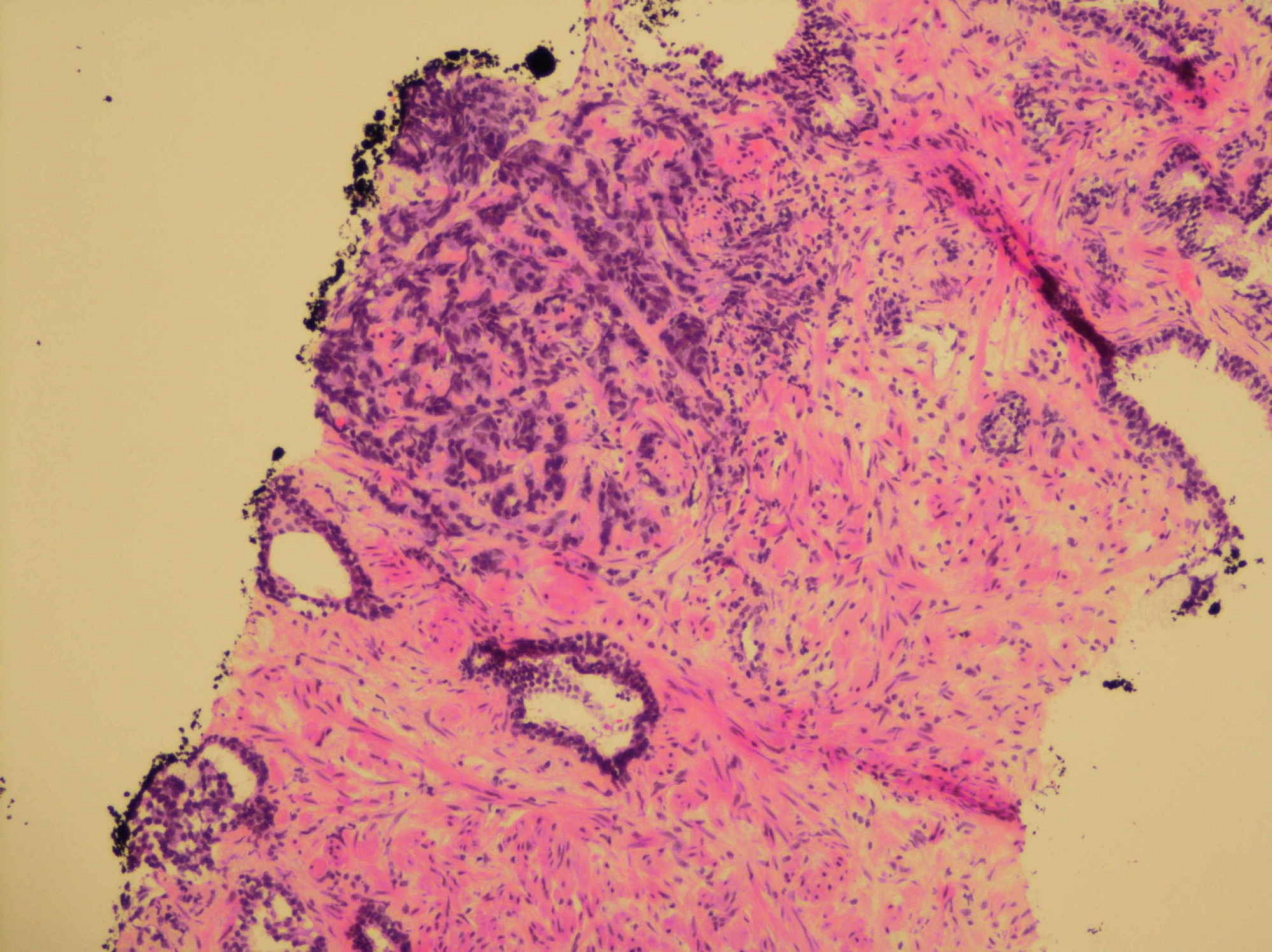
Small cell carcinoma infiltrating stroma with adjacent benign glands (100x magnification)

The patient was scheduled for a prostate biopsy. However, about two weeks after his initial visit, he presented to an outside hospital with complaints of urinary retention. He was found to have acute kidney injury from progressing bladder obstruction due to a combination of benign prostatic hyperplasia and his malignancy. A urinary Foley catheter was placed, but the patient quickly developed hematuria due to traumatic catheter insertion. When this did not resolve, he presented to this institution for further evaluation, during which time the patient denied any further subjective complaints. His kidney function eventually improved, and the patient subsequently underwent a transrectal ultrasound with a 12-core needle biopsy of the prostate. During the exam, the prostate appeared to have irregular prostatic tissue extension from the left base of the prostate. Pathology revealed small cell neuroendocrine carcinoma that was present bilaterally in the base, mid-prostate, and apex of the prostate gland (12 out of 12 cores positive) (Figures 1-3). There was evidence of perineural invasion. Immunostains showed cytokeratin and synaptophysin positivity, with a weakly positive PSA. 

**Figure 4:**
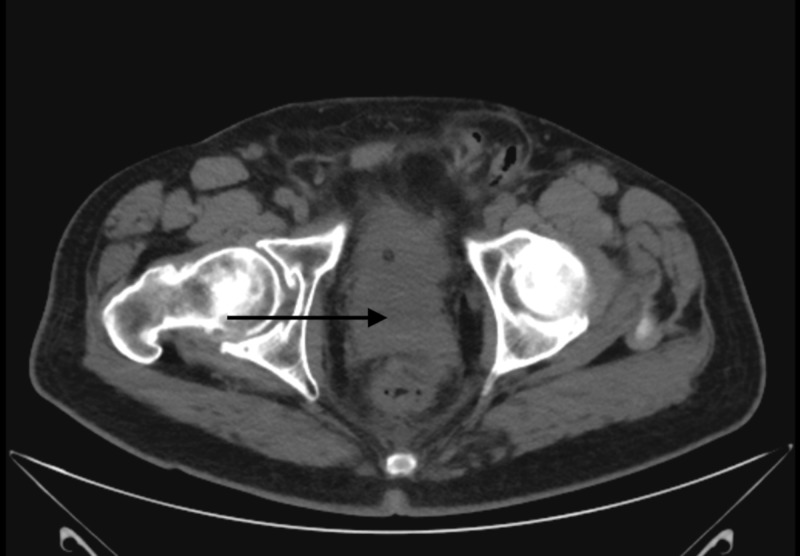
Axial view of the CT pelvis Black arrow points toward the prostate

**Figure 5:**
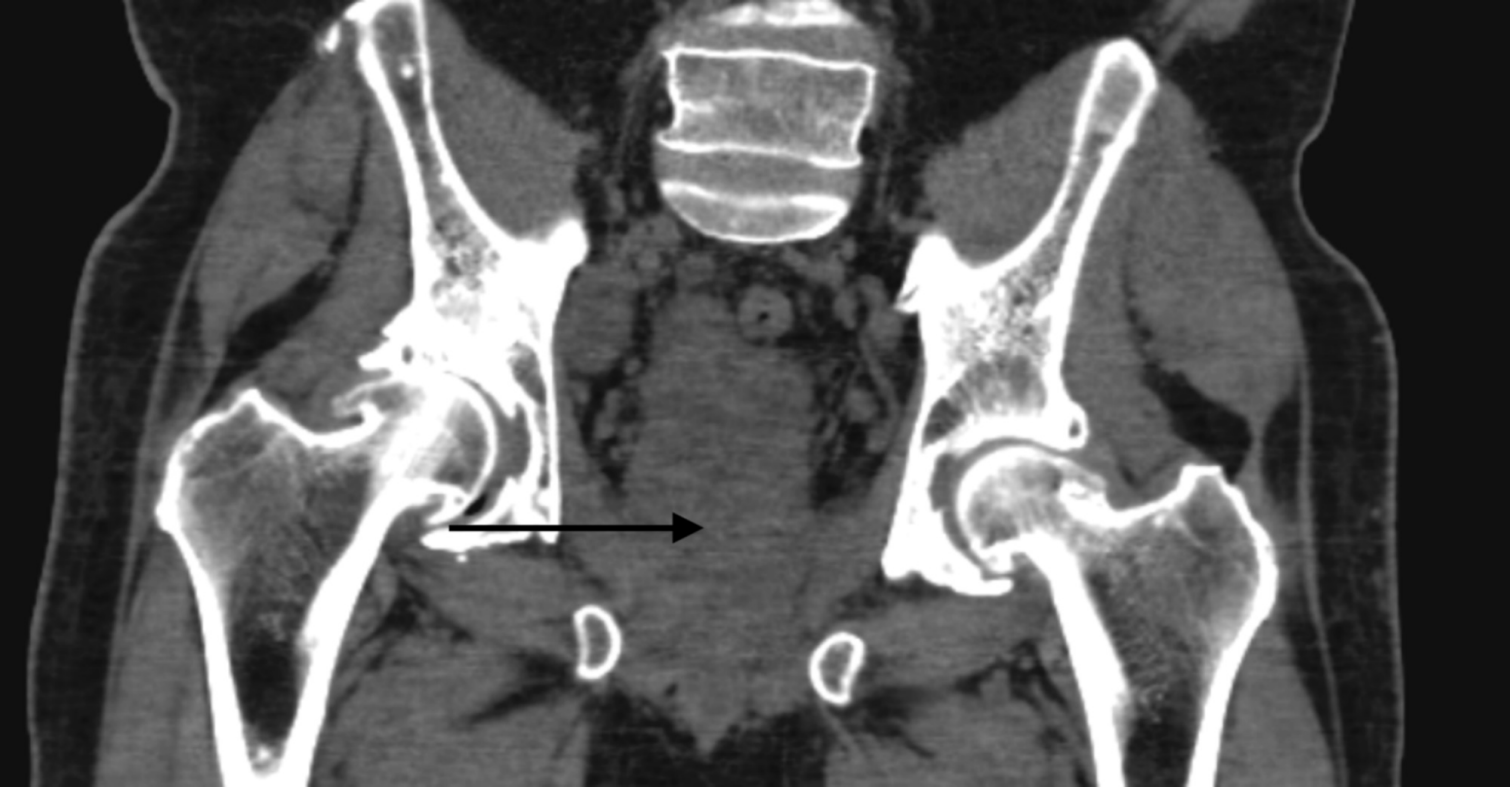
Coronal view of the CT pelvis Black arrow points toward the prostate

**Figure 6:**
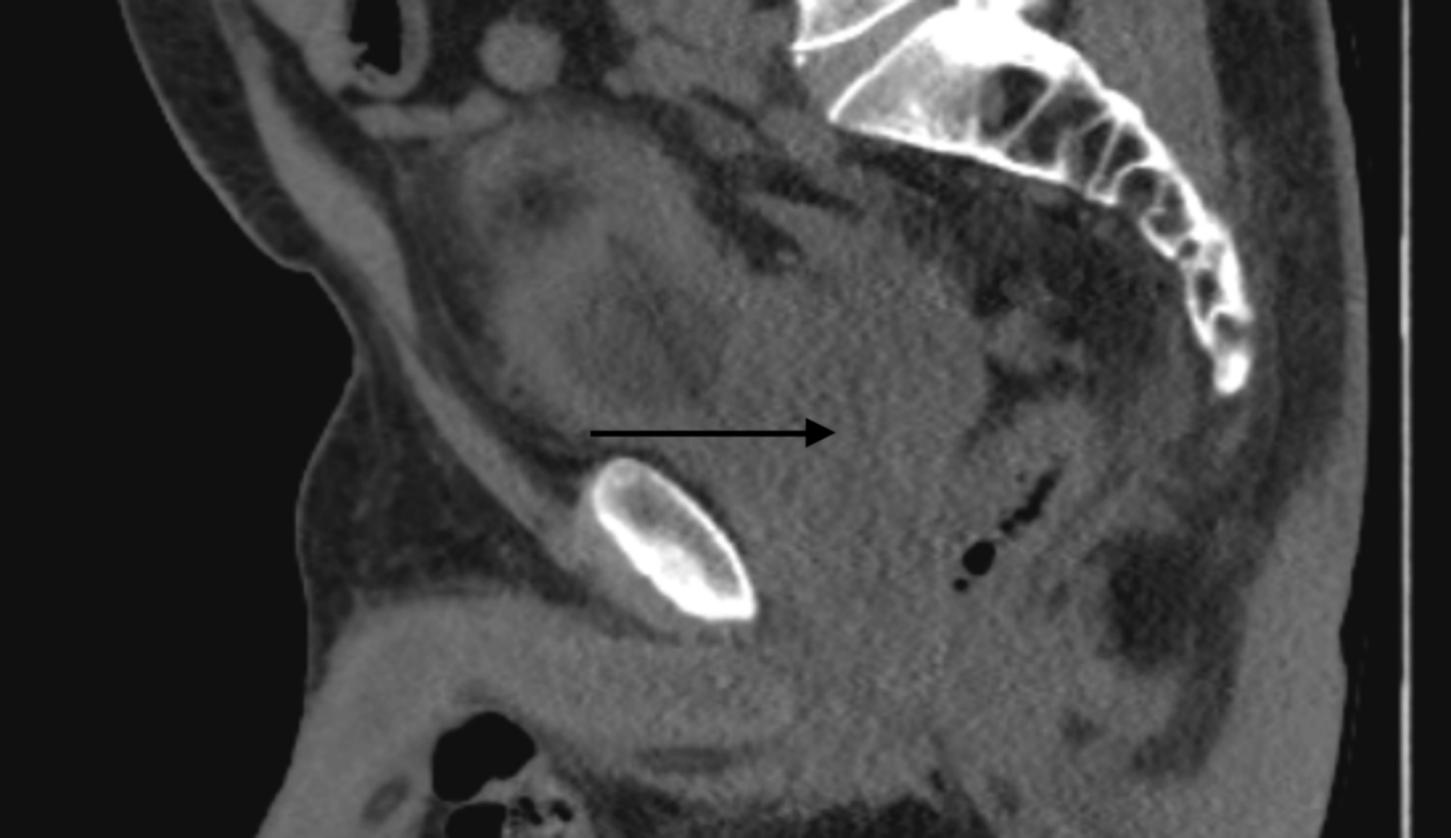
Sagital view of the CT pelvis Black arrow points toward the prostate

Shortly after his biopsy, the patient again presented to the hospital with worsening renal function. Ultrasound at this time revealed bilateral hydronephrosis. A CT of the abdomen and pelvis showed a prominent nodule in the inferior aspect of the prostate measuring 4.2 cm by 5.0 cm by 4.2 cm, which extended posterolaterally to the right (Figures 4-6). There was evidence of invasion of the left anterolateral aspect of the rectum and the bladder anteriorly. There were also two markedly enlarged right pelvic sidewall lymph nodes lateral to the external iliac vessels, measuring 2.2 cm by 3.3 cm and 1.8 cm by 2.7 cm, respectively. Besides, there was evidence of disseminated sclerotic osseous metastasis across the axial and appendicular skeleton representing prostate cancer metastasis. Lastly, there was confirmed moderate bilateral hydronephrosis with ureteral prominence. He next underwent bilateral percutaneous nephrostomy (PCN) pigtail catheter placement, and then received his first cycle of carboplatin [area under the curve (AUC) 5] with etoposide (100 mg/m2). Soon after this, the patient developed gross hematuria from his right PCN and associated anemia, all of which ended up resolving in a week. A follow-up CT of the abdomen and pelvis two weeks after his first cycle of chemotherapy showed worsening of the numerous osteoblastic lesions scattered throughout the osseous structures, which was consistent with osteoblastic metastatic disease. The right pelvic lymphadenopathy also progressed in the given interval. The patient was planned for the second cycle of chemotherapy; however, after extensive discussion with him and his family, the patient decided to pursue hospice care. The decision to opt for hospice care was made in just over three months from the time of diagnosis.

## Discussion

SCCP is an infrequent malignant entity. As such, there is a paucity of clinical data on the subject. The majority of the existing data consists of case studies and retrospective reviews. We decided to engage in a literature review with the aim of further elaborating on the most recent data, conclusions, and discoveries relating to SCCP. 

Disease characteristics

Owing to the aggressive nature of SCCP, as seen in this case, most diagnosed patients commonly present with locally invasive (bladder or bowel) disease. Around 60% of the patients are found to have metastatic disease to the lung, brain, liver, or bone (lytic lesions) at the time of diagnosis. Along these lines, poor 2- and 5- year overall survival rates (27.5 and 14.3%, respectively) have been reported in 191 patients from a Surveillance, Epidemiology, and End Results (SEER) database, with a median survival of 19 months. In this study, a multivariate analysis revealed that the strongest survival predictors included stage of the disease, the presence of mixed low-grade prostate adenocarcinoma with SCCP, and patient age. Univariate analysis showed that prostatectomy with radiation therapy, patient age of <60 years, absence of metastasis, and a mixed low-grade prostate adenocarcinoma were all favorable prognostic factors [3].

Experience has shown that, although there is a high propensity for accelerated disease progression, there is a disproportionately low-serum PSA compared to the overall burden of disease, which was also evident in this patient. Also, as seen with SCLC, SCCP can be associated with paraneoplastic syndromes. Although usually uncommon, these can include hypercalcemia, syndrome of inappropriate antidiuretic hormone production, and ectopic adrenocorticotropic hormone activity. Clinically, SCCP is linked to symptoms of accelerated disease progression [4]. The patient, in this case, presented with only an asymptomatic rise in his PSA, but then quickly progressed to develop an invasive disease with subsequent outflow obstruction and bilateral hydronephrosis, which respectively required Foley catheter insertion and PCN.

Fludeoxyglucose-positron-emission tomography (FDG-PET) scans have been found to be useful in SCCP for initial staging and treatment- response evaluation [1]. Once the condition is suspected, a biopsy should be performed to confirm the diagnosis. When presented with SCCP in the background of prostate adenocarcinoma, the specific degree of neuroendocrine differentiation discovered in the pathology has been linked with a poor prognosis, as related to the tumor aggressiveness and resulting disease progression. It has been theorized to be a result of apoptotic inhibition of prostate carcinoma cells by malignant neuroendocrine cells. On the other hand, there are distinctions between de novo SCCP morphology and prostate cancer with neuroendocrine differentiation in the background of prostate adenocarcinoma, which influences the behavior and prognosis of these two separate malignancies.

Relation to adenocarcinoma of the prostate

When SCCP is not diagnosed as a de novo primary prostate cancer, it usually reveals itself in the metastatic and castrate-resistant disease stage, when there is a progression of androgen-independent cancer that proves to be hormone therapy-resistant. Therefore, it becomes imperative to accurately detect patients with SCCP in a timely fashion, as they are unlikely to respond to subsequent androgen deprivation therapies (ADT) and carry an increased propensity for early metastasis [1].

Reports have shown that the interval between a previous diagnosis of prostate cancer and a newly discovered SCCP ranges from 1 to 300 months, with a median of 25 months [5]. Autopsy studies have also shown that men dying of castrate-resistant prostate cancer show evidence of neuroendocrine prostate cancer (NPC) in about 10-20% of the cases. Immunochemical staining has shown positive neuroendocrine carcinoma in the prostate in 20-30% of metastatic castration-resistant tumors [1,6]. However, due to the shortage of metastatic tumor biopsies pursued and the lack of appreciation of the tumor heterogeneity in SCCP, the true prevalence of this malignancy is most likely higher. 

Biochemical considerations

Within the normal prostate gland, neuroendocrine cells represent the third epithelial cell type apart from the secretory and basal cells. Their purpose is still not completely understood, but some postulated functions include controlling the secretory process in the prostate gland, involvement in the growth of the developing prostate, and eventual differentiation into a mature gland [1].

Even though the actual cellular derivation of NPC is still uncertain, the histopathological features of neuroendocrine cells closely parallel those of neuroendocrine tumors of other sites, such as the lung. For example, SCCP has shown >50% positivity in thyroid transcription factor (TTF-1) expression, 85-90% loss rate of tumor suppressor retinoblastoma protein (RB1), and 50-60% mutation rate of TP53, all similar to other primary small cell cancers [7,8]. Furthermore, similar architectural characteristics between SCCP and other small cell cancers include bustling mitotic figures, sheets of uniform cells with a high nuclear-to-cytoplasmic ratio, and diffuse infiltration consisting of poorly circumscribed margins. However, unlike in neuroendocrine tumors stemming from other sites, 60-70% of NPC tumors show evidence of ETS transcription factor family gene rearrangements, which can especially help to pinpoint NPC as the primary when a metastatic disease of unknown primary is presenting [8]. Besides, the poorly differentiated malignancies are more inclined to have a perineural invasion, another sign that the prostate gland’s innate neural and neuroendocrine tissue serves as the basis for malignant transformation into cancer. As such, the diagnosis of SCCP is confirmed based on finding such similar features of neuroendocrine malignancies found elsewhere and complemented by identifying its specific biochemical features [1]. 

Within the realm of neuroendocrine prostate tumor subtypes, there are three that have been commonly identified. The first, and the rarest, are carcinoid-like tumors. The second, and the most common one, is pure prostate adenocarcinoma with focal neuroendocrine differentiation. This concomitant neuroendocrine differentiation presents a separate cluster of cells dispersed among a more predominant collection of malignant adenocarcinoma cells. This type of mixed differentiation has been found in almost all studied prostate adenocarcinoma specimens. The third one is SCCP. The respective histopathological studies reveal that these differentiated cells (with bland nuclei and low proliferative rate) lack the androgen receptor, which indicates that their specific biological activity is independent of androgens. Not only are these malignancies nearly unresponsive to androgen-signaling inhibition, but their prognosis is not dependent on conventional pure prostate adenocarcinoma tumor stage or Gleason grade, but instead on the severity of neuroendocrine differentiation. In this case, when there is a low PSA appreciated with an unduly rapidly progression of the disease, SCCP should be highly suspected. 

As further research has been continuously progressing on these specific subtypes, there are now specific immunohistochemical biomarkers sensitive to the morphological features of SCCP and NPC that help in their detection. For example, along with the upregulation of CD56, synaptophysin, neuron-specific enolase, and chromogranin A serum-based biomarker expressions, even loss of PSA expression helps in discriminating between prostate adenocarcinoma and NPC. Besides, recent and ongoing studies have shown a diverse array of molecular markers essential to the biological foundation of NPC, which help to further distinguish NPC from small cell carcinomas of other origins. This includes necessary activation of the PI3K-Akt-mTOR pathway that helps in the formation of NPC, along with the suppressed expression of the regulator of neuronal gene expression (REST) transcription factor that acts to downregulate neuronal differentiation [9]. 

About 90% of SCCP cases show evidence of at least one neuroendocrine marker returning positive. For example, amplification of Aurora kinase A (AURKA) and N-myc (MYCN) have been used as prognostic biomarkers to help predict aggressive neuroendocrine disease progression [10]. Other molecular discoveries related to NPC include increased expression of cell cycle programs (PLK1) and chromatin modifier DEK, upregulation of EZH2 (polycomb complex gene), rearrangement of ERG gene (present in 50% of patients), loss of RB1, loss of RP54, and loss of PTEN [1,11,12]. Besides, fluorescence in situ hybridization (FISH) has shown evidence of TMPRSS2-ERG gene fusion in many SCCP cases, and cyclin D1 loss has been linked to 88% of SCCP cases compared to only 5% of prostate adenocarcinomas of Gleason score 7-10 (as evidenced by biopsies and autopsy reports) [13,14]. The role of the majority of these findings as predictive or diagnostic biomarkers remains to be demonstrated. 

Treatment

Given the increased propensity for occult metastases, localized SCCP is treated aggressively, usually with a multimodality approach. This approach is consistent with the treatment guidelines for treating limited-stage SCLC, which includes combining chemotherapy with concurrent or consolidative radiotherapy. As such, the utilization of surgery has been limited. If the patient shows aggressive clinical features of metastatic evidence, platinum-based combination chemotherapy is on the front line, again mirroring treatments for SCLC. These regimens are based on single-arm phase II studies and retrospective studies, which have included cisplatin or carboplatin with either etoposide or docetaxel versus paclitaxel [15]. Although upfront chemotherapy should be used with SCCP, the addition of ADT has shown variable results, and it depends on the specific clinical context of the case [16]. Despite this multimodality approach, disease response has been variable and usually unfavorable, especially compared to limited-stage SCLC, given the molecular complexities of these cancers. Therefore, other advanced strategies, including targeted therapies combining inhibition or manipulation of specific pathways, will be necessary for enhanced tumor control.

## Conclusions

We presented a case of locally advanced and quickly progressive SCCP. Efforts toward an enhanced understanding of the malignancy along with advancements in discoveries of the specific biochemical uniqueness are currently progressing. However, given the aggressive nature of the disease, which is similar to small cell cancers of other sites, further investigations are necessary to learn the complexities of this disease entity, so that treatment can then be better tailored to this biochemically distinctive disease process. As deducted from this specific case, obtaining biopsy confirmation and initiation of treatment are time-sensitive, as a low PSA level can be misleading and may belie the aggressive clinical nature of the disease process.
